# Genome-wide association mapping of resistant starch for improved nutritional traits in lentils

**DOI:** 10.3389/fpls.2026.1937124

**Published:** 2026-10-30

**Authors:** Hatice Sari, Dorrie Main, Yu Ma, Sean Finnie, Zhiwu Zhang, Sintayehu Daba, Ping Zheng, Stephen Ficklin, Adrian Russell, Briar Kinney, Rebecca J. McGee

**Affiliations:** 1 Department of Horticulture, Washington State University, Pullman, WA, United States; 2 Department of Horticulture and Crop Science, Ohio State University, Columbus, OH, United States; 3 School of Food Science, Washington State University, Pullman, WA, United States; 4 Department of Crop and Soil Sciences, Washington State University, Pullman, WA, United States; 5 Washington State University, Northwestern Washington Research & Extension Center, Mount Vernon, WA, United States; 6 Plant Research (NZ) Ltd., Templeton, Irwell, New Zealand

**Keywords:** candidate genes, GWAS, *Lens culinaris*, Lentil, nutrition, resistant starch

## Abstract

**Introduction:**

Lentil (*Lens culinaris Medik*.) is a nutrient-dense legume that plays a critical role in global food security and human health. In addition to being a rich source of protein, fiber, and micronutrients, lentil contains resistant starch (RS), a component of dietary fiber associated with improved glycemic response, enhanced gut microbiota, and reduced inflammation. However, the genetic basis of RS variation in lentil remains poorly understood.

**Methods:**

Resistant starch concentration, total starch concentration, 100-seed weight, and plot seed weight were quantified in a panel of 192 diverse lentil accessions and five checks evaluated across three environments. Genome-wide association studies (GWAS) were conducted using 5,583 high-quality single nucleotide polymorphisms (SNPs). Putative candidate genes were identified within 100-kb windows of significant marker -trait associations (MTAs), followed by gene ontology (GO) and Kyoto Encyclopedia of Genes and Genomes (KEGG) pathway enrichment analyses.

**Results:**

Nine significant MTAs were identified for RS, three for total starch, 20 for 100-seed weight, and 11 for plot seed weight, explaining 2.9%–42.6% of the phenotypic variance. Putative candidate genes included soluble starch synthase III (SS3), which is involved in starch biosynthesis. Functional enrichment analyses revealed overrepresentation of biological processes associated with oxidative stress response, detoxification, starch and amylopectin biosynthesis, ATP synthesis, and auxin transport. Enriched molecular functions included peroxidase, peptidyl -prolyl isomerase, and UDP-glucosyltransferase activities, while enriched cellular components included the chloroplast, peroxisome, vacuole, and cell junctions.

**Discussion:**

These findings provide insights into the genetic basis of resistant starch accumulation in lentil seeds and identify potential genetic targets for future breeding efforts aimed at improving the nutritional quality of lentil cultivars.

## 1 Introduction

Lentils (*Lens culinaris* Medik.) are one of the most nutritionally valuable, globally cultivated pulse crops. As a self-pollinating, cool-season annual legume, lentil was among the first domesticated plant species and remains a dietary staple in many regions (Coyne and McGee, 2013). According to the Food and Agriculture Organization (FAO), global lentil production reached approximately 7.9 million tons in 2024 (FAOSTAT, 2026), highlighting their continued importance in global food systems. Lentils serve as a crucial source of plant-based protein and contribute significantly to reducing malnutrition, particularly in low-income countries where protein deficiency is widespread (Kumar et al., 2022; Aski et al., 2023, Sari et al., 2024a). Over the past 25 years, lentils have attracted growing interest due to their nutritional density and environmental sustainability, being rich in protein, dietary fiber, essential minerals, and health-promoting phytochemicals (Johnson et al., 2013; Romano et al., 2021; Dhull et al., 2023; Kaale et al., 2023; Li et al., 2024). Notably, lentils also contain substantial amounts of resistant starch, a physiologically functional component of dietary fiber (Chen et al., 2016; Okumus et al., 2018; Johnson et al., 2021).

Resistant starch (RS) is a unique form of starch that escapes digestion in the small intestine and undergoes fermentation in the colon by the gut microbiota, producing short-chain fatty acids (SCFAs), particularly butyrate (Englyst et al., 1987; Brouns et al., 2002; Chen et al., 2024). RS is generally classified into five types based on the mechanisms underlying its resistance to enzymatic digestion (Raigond et al., 2015; Lockyer and Nugent, 2017). RS1 consists of starch physically protected by intact cell walls or protein matrices, whereas RS2 is resistant due to its native crystalline structure. RS3, commonly referred to as retrograded starch, forms when cooked starchy foods are cooled, resulting in the reorganization of amylopectin chains into structures that are less susceptible to enzymatic hydrolysis (Ma et al., 2020). RS4 includes chemically modified starches produced through processes such as cross-linking, etherification, or esterification, while RS5 consists primarily of amylose–lipid complexes and resistant maltodextrins formed through structural rearrangements during processing. Despite their structural differences, all resistant starch types can contribute to fermentation in the colon and subsequent SCFA production. These SCFAs exert multiple beneficial effects, including improved glycemic control, enhanced insulin sensitivity, reduced systemic inflammation, increased satiety, and support for a healthy gut microbiome (Sajilata et al., 2006; Bindels et al., 2017). Among these, butyrate plays a key role in maintaining colonic health by serving as the primary energy source for colonocytes and by modulating cellular processes that reduce inflammation and may lower the risk of colorectal cancer (Hylla et al., 1998; Grubben et al., 2001). In addition to its role in colonic health, resistant starch supports blood glucose management by slowing starch digestion and glucose release, thereby lowering the glycemic index of foods. This effect helps to moderate postprandial blood sugar and insulin levels, which is particularly beneficial for individuals with type 2 diabetes or those at risk of metabolic disorders (Kim et al., 2024). Resistant starch also exhibits prebiotic properties, as it reaches the colon intact and serves as a fermentable substrate for beneficial bacteria populations such as *Bifidobacterium* and *Lactobacillus*, promoting gut microbial balance and immune function (Fuentes‐Zaragoza et al., 2011). Due to these wide-ranging health benefits, resistant starch is increasingly recognized as both a key component of dietary fiber and a target trait for improving the nutritional quality of staple crops through breeding and food-based strategies. As global demand for functional foods continues to rise, improving the nutritional profile of staple crops like lentils has become a priority for plant breeding programs (Nugent, 2005; Ma et al., 2018; Okumus et al., 2018; Li et al., 2019).

Lentils are naturally rich in protein, dietary fiber, essential micronutrients, and phytochemicals, positioning them as a key component of healthy diets (Urbano et al., 2007; Coyne and McGee, 2013; Sen Gupta et al., 2013; Dhull et al., 2023). Among these attributes, lentils are also a source of resistant starch, a form of dietary fiber associated with improved glycemic response, gut microbiota modulation, and reduced inflammation. Reported RS concentrations in lentils range from approximately 4% to 21% of total starch, depending on genotype, seed type, and processing method (Garcia-Alonso et al., 1998; Tovar et al., 1992; Ma et al., 2018; Okumus et al., 2018). Resistant starch has been more extensively studied in other crops such as rice (*Oryza sativa* L.), wheat (*Triticum aestivum* L.), and barley (*Hordeum vulgare* L.), where multiple gene families including starch synthases, branching enzymes, and transporters have been linked to RS variation through mutant screens and quantitative trait loci (QTL) analyses (Shu et al., 2012; Bao et al., 2017; Zhang et al., 2020; To et al., 2025). However, the genetic basis of resistant starch accumulation in lentils remains largely unexplored.

Although *L. culinaris* is a diploid species (2n = 14), its large genome (~4 Gb) is structurally complex (Ramsay et al., 2021; Tayşi et al., 2022). The development of high-throughput genotyping and statistical tools has enabled the application of genome-wide association studies (GWASs), a powerful approach to dissect complex quantitative traits in plants (Tian et al., 2020). GWAS evaluates the association between individual genetic markers and phenotypic variation across a diverse population. Initially developed in human genetics (Hindorff et al., 2009), GWAS is now widely applied in model organisms and crops, offering high-resolution mapping without the need for biparental mapping populations (Korte and Farlow, 2013). GWAS facilitates the identification of genomic regions and candidate genes linked to traits of interest by leveraging natural variation (Korte and Farlow, 2013; Mountjoy et al., 2021). This high-resolution approach is especially valuable for exploring complex quantitative traits like resistant starch, which are influenced by multiple genes and environmental interactions (Zhang et al., 2019; Wang et al., 2020). In pulse crops, GWAS has already been successfully used to uncover genetic loci associated with abiotic/biotic resistances (Ma et al., 2020; Dissanayake et al., 2021; Gela et al., 2021), seed nutrients (Mugabe et al., 2023; Sari et al., 2024b; Sari et al., 2025; Uhdre et al., 2025), and agronomic traits (Rajendran et al., 2021; Balech et al., 2024). However, the use of GWAS to dissect RS concentration is still in its infancy (Johnson et al., 2021), with only a few studies, including in common bean (*Phaseolus vulgaris* L.), pea (*Pisum sativum* L.), chickpea (*Cicer arietinum* L.) and soybean (*Glycine max* (L.) Merr.), on starch-related traits (Pradhan et al., 2014; Carpenter et al., 2017; Dhungana et al., 2017; Gali et al., 2019; Bassett et al., 2021; Mendes et al., 2021). Applying this approach to resistant starch in lentils can offer novel insights into the biological pathways involved and facilitate the development of even more nutrient-rich cultivars through marker-assisted selection.

In this context, the objectives of this study were to (i) evaluate natural variation in resistant starch and total starch concentrations across a diverse lentil panel; (ii) identify genomic regions associated with resistant starch, total starch, 100-seed weight, and plot seed weight using GWAS; and (iii) identify putative candidate genes contributing to resistant starch accumulation. The findings advance our understanding of the genetic architecture of resistant starch in lentils and provide valuable genomic resources to support marker-assisted breeding for improved nutritional quality in this globally important crop.

## 2 Materials and methods

### 2.1 Plant materials and experimental traits

In this study, a total of 192 lentil accessions from the Lentil Single Plant (LSP) collection, obtained from the Western Regional Plant Introduction Station (USDA-ARS, Pullman, WA), along with well-known varieties of lentils commonly grown in the Palouse region five check cultivars, Avondale, Cedar, Crimson, Merritt, and Pardina, were evaluated (**Supplementary Table 1**). The field trials were conducted using an augmented experimental design across three environments under irrigated conditions: Spillman, WA, USA (46°42′01.1″ N; 117°08′41.4″ W); Central Ferry, WA, USA (46°39′5.1″ N; 117°45′45.4″ W); and Irwell, Canterbury, NZ (43°43′17.9″S, 172°21′1.4″E). At Irwell, the experiment was planted in October 2023 and harvested in March 2024, whereas the experiments at Spillman and Central Ferry were planted in April 2024 and harvested in August 2024. At the Spillman and Central Ferry sites, the experiment was arranged in 10 blocks, while at the Irwell site, it was organized in five blocks. The check lines were replicated in each block, while the LSP accessions were planted once. For each accession, 60 seeds were sown in double rows. At Spillman, a post-plant, pre-emergence application of Metribuzin (4-Amino-6-(1,1-dimethylethyl)-3-(methylthio)-1,2,4-triazin-5(4*H*)-one), 219.42 ml/ha, and Linuron (3-(3,4 dichlorophenyl)-1-methoxy-1-methylurea), 700.51 g/ha was applied. A pre-emergence herbicide, S-metolachlor (2-chloro-N-(2-ethyl-6-methylphenyl)-N-[(1S)-2-methoxy-1-methylethyl] acetamide), was applied at Central Ferry at a rate of 1,170 ml/ha; no fertilizers or additional herbicides were applied during the growing season. Weeds were managed manually throughout the growing season. At maturity, each plot was hand-harvested, followed by uniform drying to ensure consistent seed moisture levels. Threshing was performed using a Vogel thresher, and final seed cleaning was conducted with a seed blower.

### 2.2 Resistant and digestible starch analysis

The concentrations of resistant (RS), digestible (DS), and total starch (TS) were measured using Megazyme’s (Bray, Ireland) Resistant Starch (Rapid) Assay Kit with an updated protocol based on McCleary et al. (2002), corresponding to AOAC Method 2002.02 and AACC Method 32-40.01. To ensure analytical quality and assay consistency, the Megazyme Resistant Starch Control Flour (certified RS concentration: 47.4%) was included in every assay batch and analyzed in duplicate. The measured values of the control flour were monitored throughout the study and remained within the manufacturer’s acceptable range, confirming consistent assay performance across batches. The measured RS values of the control flour were monitored throughout the study to verify assay performance and reproducibility. According to the manufacturer’s validation data, the method is applicable to samples containing more than 2% resistant starch (w/w) and routinely achieves a standard error of approximately ±5% for samples within this concentration range. Since all lentil samples contained RS concentrations well above 2%, the assay operated within its validated analytical range.

Lentil seeds from each accession were ground into fine flour using a stainless-steel coffee grinder. A 100-mg sample of the flour was weighed into a 15-ml falcon tube. To each tube, 0.2 ml of enzyme solution (pancreatic α-amylase/ammonium sulfate suspension) and 3.8 ml of sodium maleate buffer (50 mM, pH 6.0) containing 2 mM calcium chloride dihydrate were added. Tubes were vortexed thoroughly and incubated at 37 °C for 4 hours with continuous circular shaking (200 strokes per minute). Following incubation, 4 ml of 95% ethanol was added to each tube to precipitate resistant starch. The contents were vortexed, centrifuged, and decanted into a 100-ml volumetric flask. Two additional washes were performed using 2 and 6 ml of 50% ethanol, respectively. After each wash, the pellet was vortexed, centrifuged, and decanted. The supernatants from all washes were pooled and diluted to 100 ml with distilled water for digestible starch measurement.

The remaining pellet, containing resistant starch, was dissolved in 2 ml of 1.7 M NaOH while stirring on a magnetic stirrer in an ice-water bath for 20 minutes. Next, 8 ml of sodium acetate buffer (1.0 M, pH 3.8) containing 5 mM calcium chloride was added, followed immediately by 0.1 ml of amyloglucosidase (AMG, 3,300 U/ml). Tubes were incubated at 50 °C in a water bath for 30 minutes, followed by vortex for 10 seconds. Following vortex, the samples were diluted to 100 ml with distilled water. For quantification, 0.1 ml of the RS solution was mixed with 3 ml of glucose oxidase/peroxidase (GOPOD) reagent in duplicate tubes and incubated at 50 °C for 20 minutes. A glucose standard (1 mg/ml in 0.2% benzoic acid) and reagent blanks were included in each batch. To determine digestible starch, 0.1 ml of the supernatant (from the pooled ethanol washes) was treated with 0.1 ml of diluted AMG (110 U/ml) and incubated at 50 °C for 30 minutes. After incubation, 3 ml of GOPOD reagent was added, and the tubes were incubated at 50 °C for 20 minutes.

Absorbance was measured at 510 nm using a Genesys 50 UV–Visible Spectrophotometer (Thermo Fisher Scientific, part no. 840-298000). Resistant and digestible starch concentrations were calculated using the following equations:


$$RS\;\left(\frac{\text{g}}{100}\text{g}\right)=\frac{\left(\text{ΔA}\times \text{ F}\times \text{ EV}\right)}{W\times 0.9}\;,$$

$$DS\;\left(\frac{\text{g}}{100}\text{g}\right)=\frac{\left(\text{ΔA}\times \text{ F}\times \text{ EV}\right)}{W\times 0.9}\;,$$

$$Total\;Starch\;\left(\frac{\text{g}}{100}\text{g}\right)=\text{RS}+\text{DS },$$

Where: ΔA = absorbance of the sample solution (against reagent blank), F = factor to convert absorbance to µg glucose (derived from 100 µg standard), EV = extraction volume (10.3 ml or 100 ml depending on assay step), W = sample weight (mg), and 0.90 = conversion factor for free glucose to anhydroglucose (162/180). These calculations were facilitated using Mega-Calc™ data calculator [Rapid Resistant Starch (K-RAPRS)].

### 2.3 Statistical analysis

For all traits, the mean, standard error, and range were calculated for each accession across environments. The distributions of RS, TS, 100-SW, and Plot-SW were visualized using boxplots to assess trait variability and data spread at each location. Descriptive statistics and boxplots were generated using Microsoft Excel (Version 16.110.3). Phenotypic data for RS, TS, 100-SW, and Plot-SW were analyzed using linear mixed models fitted by restricted maximum likelihood (REML) in the lme4 package in R (Bates et al., 2015). The following model was fitted separately for each trait:


$$y_{ijk}=\mu +E_{j}+G_{i}+{\left(GE\right)}_{ij}+B_{k\left(j\right)}+\epsilon_{ijk}$$

where $y_{ijk}$ is the observed phenotypic value of genotype i. in environment j and block k; μ is the overall mean; $E_{j}$ is the fixed effect of environment; $G_{i}$ is the random effect of genotype; ${\left(GE\right)}_{ij}$ is the random genotype × environment interaction; $B_{k\left(j\right)}$ is the random effect of block nested within environment; and $\epsilon_{ijk}$ is the residual error. The significance of the fixed effect of environment was evaluated using F-tests with Satterthwaite’s approximation for denominator degrees of freedom implemented in the lmerTest package (Kuznetsova et al., 2017). The significance of the random effects was assessed using likelihood-ratio tests by comparing models with and without each random effect. Best linear unbiased predictors (BLUPs) for genotype were extracted from the fitted mixed models using the ranef () function in the lme4 package. Genotype BLUPs for RS and TS, adjusted for environmental effects, block effects, and genotype × environment interaction, were used as the phenotypic values for genome-wide association analysis.

Generalized broad-sense heritability (H²) was estimated across environments using the method of Cullis et al. (2006). The linear mixed model was fitted using the lme4 package in R and included environment as a fixed effect and genotype, block nested within environment, and G×E interaction as random effects. Cullis generalized broad-sense heritability was calculated using the ‘H^2^_Cullis’ function implemented in the heritable R package as


$$H_{Cullis}^{2}=1-\;\frac{{\bar{PEV}}_{\text{Δ}}}{2\sigma_{G}^{2}}$$

where $\sigma_{G}^{2}\;$ is the estimated genotypic variance and ${\bar{PEV}}_{\text{Δ}}$ is the mean prediction error variance of pairwise differences among genotype BLUPs. This approach accounts for differences in the precision of genotype predictions and is therefore suitable for the unbalanced structure of the augmented experimental design.

### 2.4 Correlation analysis

Lentil seed coat colors were determined using the QM3i VIBE imaging analytics instrument (Vibe Imaging Analytics Inc. Capitola, CA). To explore relationships among seed coat color traits and key agronomic and nutritional variables, Pearson’s correlation coefficients were calculated for RS, TS, 100-SW, Plot-SW, and seed coat color percentages (CP)—OrangeCP, GreenCP, BlackCP, BrownCP, and PinkCP. Correlation matrices were generated using the cor() function in R (R Core Team, 2024) based on pairwise complete observations. Statistical significance of each pairwise correlation was assessed using the cor_pmat() function from the ggcorrplot package (version 0.1.3; Kassambara, 2019), which provides corresponding p-values.

The correlation matrix was visualized with the ggcorrplot() function, displaying both the correlation coefficients and a color gradient (red: negative correlation; black: positive correlation; white: no correlation). Only statistically significant correlations (*p* ≤ 0.05) are colored and labeled; non-significant correlations are left blank. All analyses and visualizations were performed in R.

### 2.5 Genotyping and genome-wide associations

Previously generated genotyping-by-sequencing (GBS) data were used for genome-wide association analysis (GWAS) (Ma et al., 2020). As described by Ma et al. (2020), GBS libraries were prepared using a two-enzyme approach with *PstI* and *MspI* (Poland et al., 2012), followed by ligation with 4–9 bp barcode adapters. Barcoded samples were pooled, cleaned with AMPure XP beads, PCR-amplified, and sequenced on an Illumina HiSeq™ 2500 platform (single-end, 100 bp) at Washington State University’s Genomics Core Laboratory. Reads were demultiplexed, trimmed to 80 bp using process_radtags in Stacks v2.0 (Catchen et al., 2013), and aligned to the lentil reference genome (Ramsay et al., 2021) with Burrows-Wheeler Aligner (BWA). Multi-mapped reads were removed using SAMtools (Li, 2011), and SNPs were called using the ref_map.pl pipeline in STACKS. A total of 62,606 raw SNPs were identified and filtered using VCFtools (v0.1.16; Danecek et al., 2011) to retain only biallelic SNPs with a minor allele frequency (MAF) > 0.05 and ≤50% missing values (**Supplementary Figure 1**). No additional filtering based on heterozygosity was applied during SNP filtering. SNPs located on unanchored contigs were subsequently removed, resulting in a final dataset of 5,583 high-quality SNPs distributed across the seven lentil chromosomes for genome-wide association analysis.

Population structure was evaluated using principal component analysis (PCA), and the first three principal components were included as covariates in the GWAS. GWAS was performed to identify SNP markers associated with RS and TS concentrations, 100-SW, and Plot-SW. This involved identifying marker–trait associations (MTAs) using the BLINK (Bayesian-information and Linkage-disequilibrium Iteratively Nested Keyway) model implemented in the GAPIT 3.0 R package (Huang et al., 2019; Wang and Zhang, 2021). The BLINK model employs a fixed-effect approach that integrates Bayesian information to reduce both false positives and false negatives. It also leverages linkage disequilibrium (LD) structure, thereby eliminating the assumption that markers must be evenly distributed across the genome (Huang et al., 2019). To determine statistically significant associations, a Bonferroni correction [ $-\log_{10}\left(0.05\;÷number\;of\;markers\right)$] was applied to control for multiple testing, and SNPs exceeding this threshold were considered significantly associated with the trait.

### 2.6 Linkage disequilibrium, candidate genes, functional annotation, and KEGG enrichment analysis

Linkage disequilibrium (LD) analysis was conducted using TASSEL v5.2.96 (Bradbury et al., 2007). LD decay was subsequently estimated in R, following the formula described by Remington et al. (2001). The expected value of LD, denoted as *E(r²)* under drift–recombination equilibrium, was calculated as


$$E\left(r^{2}\right)=\frac{\left(10+C\right)}{\left(2+C\right)\left(11+C\right)}\;\times \;\left[1+\frac{\left(3+C\right)\left(12+12C+C^{2}\right)}{n\;\left(2+C\right)\left(11+C\right)}\right],\;$$

where *N* is the effective population size, *c* is the recombination fraction between loci, and *C* is calculated as *4Nc*.

An LD window of 100 kb was utilized to identify candidate genes associated with resistant starch. Gene models were determined using the PulseDB platform (https://www.pulsedb.org/jbrowses) for the *Lens culinaris* cv. CDC Redberry v2.0 genome (Ramsay et al., 2021). To infer putative gene functions, all candidate lentil protein sequences were queried against *Arabidopsis thaliana* Araport11 proteome (release 2022-09) database using BLASTP (BLAST+ v2.12.0). Searches were conducted using an E-value cutoff of 1 × 10–^6^ to retain high-confidence matches. For each protein, up to the top 10 BLASTP hits were retained to support functional annotation, resulting in a total of 410 homolog hits (**Supplementary Table 2**).

Gene ontology (GO) and KEGG pathway enrichment analyses were performed using ShinyGO v0.85.1 (release 2025-09; Ge et al., 2020). Only annotated *Arabidopsis* homologs were included in the analysis. Statistical significance was evaluated using false discovery rate (FDR)-adjusted *P*-values (Benjamini–Hochberg), and KEGG pathways with FDR< 0.05 were considered significantly enriched. Enrichment results were visualized as bubble plots in R (2025.09.1 + 401) using the tidyverse (Wickham et al., 2019) and ggrepel (Slowikowski et al., 2021) packages.

To determine the genomic locations and predicted consequences of significant MTAs, SNPs were annotated using the *Lens culinaris* cv. CDC Redberry v2.0 reference genome sequence (FASTA) and its corresponding gene annotation file (GFF3) (Ramsay et al., 2021). Allelic information for each significant SNP was obtained from the filtered HapMap genotype file used for GWAS. SNP positions were matched to the reference genome, and the reference nucleotide was verified directly from the genome sequence; the alternative allele was then determined from the alleles reported in the HapMap file. Genomic coordinates in the GFF3 file were used to determine whether each SNP was located within a coding sequence (CDS), intron, untranslated region (UTR), or intergenic region. For SNPs located within CDS regions, CDS segments belonging to the corresponding transcript were ordered according to the annotated strand orientation and used to reconstruct the coding sequence. The position of each SNP within the coding sequence and codon was then determined. For genes on the negative strand, alleles were converted to their complementary bases to represent the coding strand. Reference and alternative codons were translated using the standard genetic code, and their predicted amino acid sequences were compared. Coding SNPs were classified as synonymous when the amino acid remained unchanged, nonsynonymous when the amino acid changed, or stop-gained when the alternative allele introduced a premature stop codon. Sequence extraction, reverse-complementation, and codon translation were performed in R using the Biostrings package.

## 3 Results

### 3.1 Phenotypic traits

Descriptive statistics of agronomic and starch traits measured in the lentil panel across three environments are summarized in **Table 1**. Boxplot comparisons across environments revealed substantial variation in all four traits (**Figure 1**). Resistant starch concentrations varied across environments, with median values highest in Spillman, followed by Central Ferry and Irwell (**Figure 1a**). Total starch concentrations in lentil seeds showed narrower variation across environments compared to resistant starch. Boxplot analysis revealed similar mean total starch values among Irwell, Spillman, and Central Ferry environments, with only modest shifts in distribution (**Figure 1b**). The 100-SW showed notable variation among environments (**Figure 1c**). Irwell exhibited the highest median and broader range of seed weights, while Spillman had the lowest median values and a narrower distribution. Central Ferry presented an intermediate median but also displayed a wide distribution in values. Plot-SW showed substantial variation across environments. Irwell had the highest variation. Mean values were higher at Irwell and Spillman compared to Central Ferry (**Figure 1d**). These results indicate considerable environmental influence on starch composition and yield-related traits within the lentil panel. Mixed-model analysis revealed significant environmental effects for RS, TS, 100-SW, and Plot-SW (**Supplementary Table 3**). Block effects nested within environment were also significant for all four traits, indicating substantial within-environment field variation. Generalized broad-sense heritability varied considerably among traits. Resistant starch showed moderate-to-high heritability (H² = 0.632), whereas total starch had low heritability (H² = 0.182). Heritability was very high for 100-seed weight (H² = 0.987) and moderate for plot seed weight (H² = 0.440) (**Supplementary Table 3**).

**Table 1:** Descriptive statistics for resistant starch (RS), total starch (TS), 100-seed weight (100-SW), and plot seed weight (Plot-SW) in the lentil panel across three environments (Irwell, Spillman, and Central Ferry, WA).

Traits	Irwell			Spillman, WA			Central Ferry, WA		
	Min–Max	Mean	SE	Min–Max	Mean	SE	Min–Max	Mean	SE
RS (%)	7.3–27.8	15.7	0.3	8.5–28	19.4	0.3	8.4–23.6	16.3	0.2
TS (%)	31.6–49.8	42.3	0.2	34.2–53.8	41.3	0.2	31.1–47.1	39.6	0.2
100-SW (g)	2.1–7.5	3.8	0.1	1.6–6.3	3.1	0.1	2–6.9	3.7	0.1
Plot-SW (g)	29.6–529.4	173.3	7	41.1–263.2	160.3	3.3	18.0–190.8	96.4	2.8

**Figure 1:**
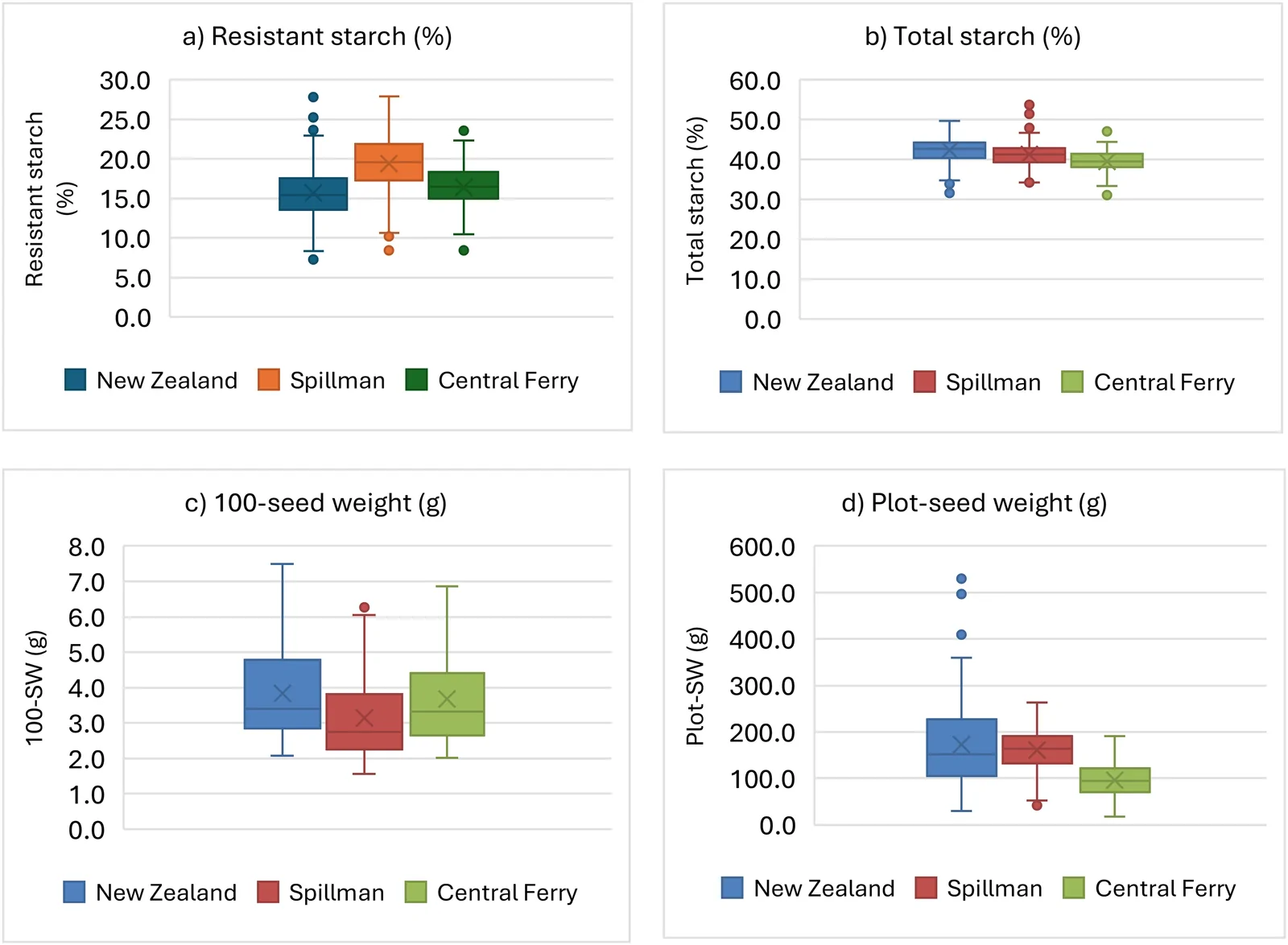
Boxplots of four traits evaluated in the LSP panel across three environments: **(a)** resistant starch (%), **(b)** total starch (%), **(c)** 100-seed weight (g) (100-SW), and **(d)** plot-seed weight (g) (Plot-SW). Each box represents the interquartile range (IQR), the line within each box indicates the median, and whiskers extend to 1.5× IQR. Outliers are shown as individual points. Data are shown for three environments: Irwell (blue), Spillman (orange), and Central Ferry (green).

### 3.2 Correlation analyses

Correlation analysis among resistant starch and seed coat color traits (OrangeCP, GreenCP, BlackCP, BrownCP, and PinkCP) revealed several significant correlations in both the Spillman and Central Ferry environments (**Figure 2**). In both environments, RS exhibited moderate negative correlations with OrangeCP (r = –0.33 in Spillman; r = –0.44 in Central Ferry), indicating that higher orange count percentages were associated with lower resistant starch concentration. Similarly, RS showed negative correlation with PinkCP (r = –0.40 in Spillman; r = –0.39 in Central Ferry). BrownCP and BlackCP were positively correlated with RS in Spillman (r = 0.28, r = 0.2), and in Central Ferry (r = 0.14, r = 0.17), respectively. Strong positive correlations were observed between BlackCP and BrownCP, as well as PinkCP and OrangeCP. The correlation structure was consistent across environments, highlighting stable negative correlations between RS and PinkCP–OrangeCP, and positive correlations between RS and BlackCP–BrownCP.

**Figure 2:**
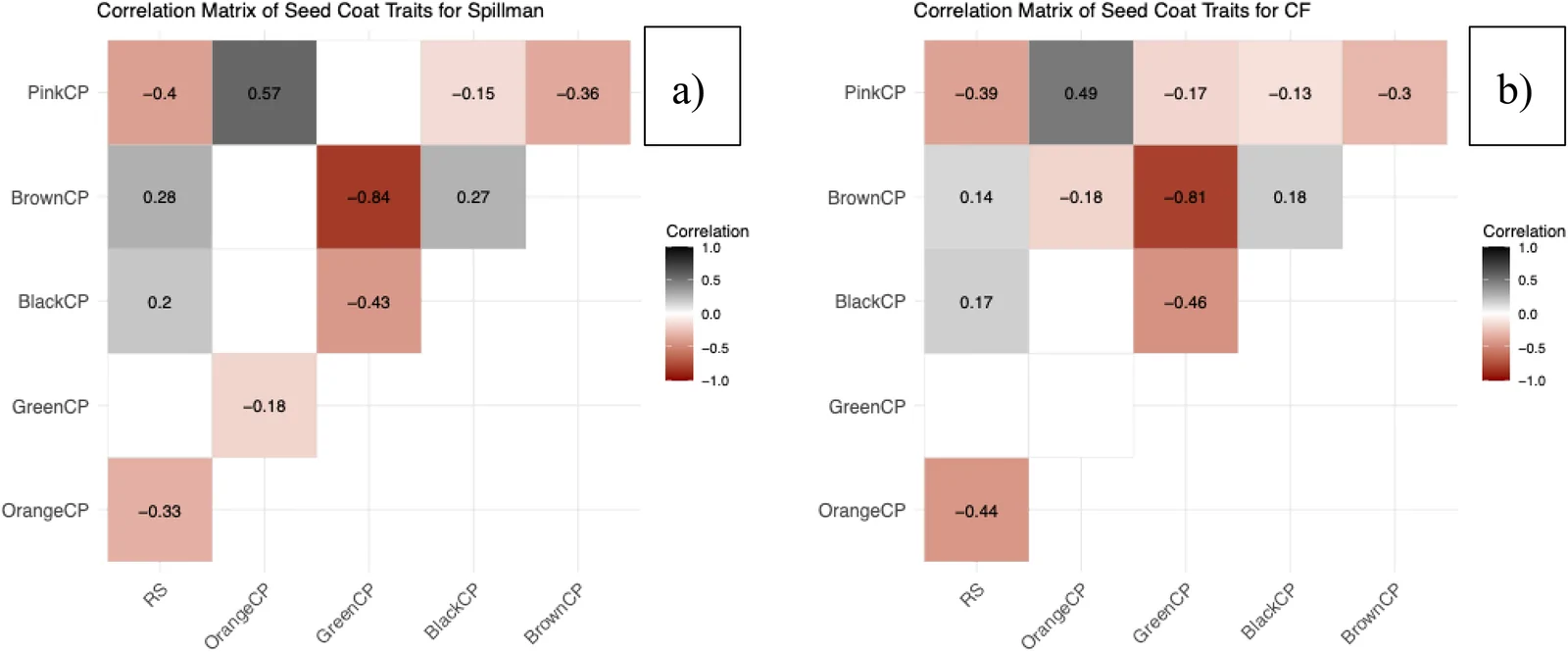
Correlation matrix of seed coat color traits and resistant starch (RS) in lentils. CP is seed coat color percentages. The color scale represents the strength and direction of Pearson correlations with black representing positive correlations, red representing negative correlations, and white indicating no correlation. Values shown are Pearson correlation coefficients. Only statistically significant correlations (p ≤ 0.05) are colored and labeled; non-significant correlations are left blank. **(a)** Spillman environment; **(b)** Central Ferry environment.

The correlation analysis revealed significant correlations among resistant starch, total starch, 100-SW, and Plot-SW. RS showed a positive correlation with TS (r = 0.37) and Plot-SW (r = 0.23), indicating that higher RS concentration is generally associated with greater starch accumulation and yield (**Figure 3**). 100-SW showed weak negative correlations with RS (r = -0.34) and TS (r = -0.19).

**Figure 3:**
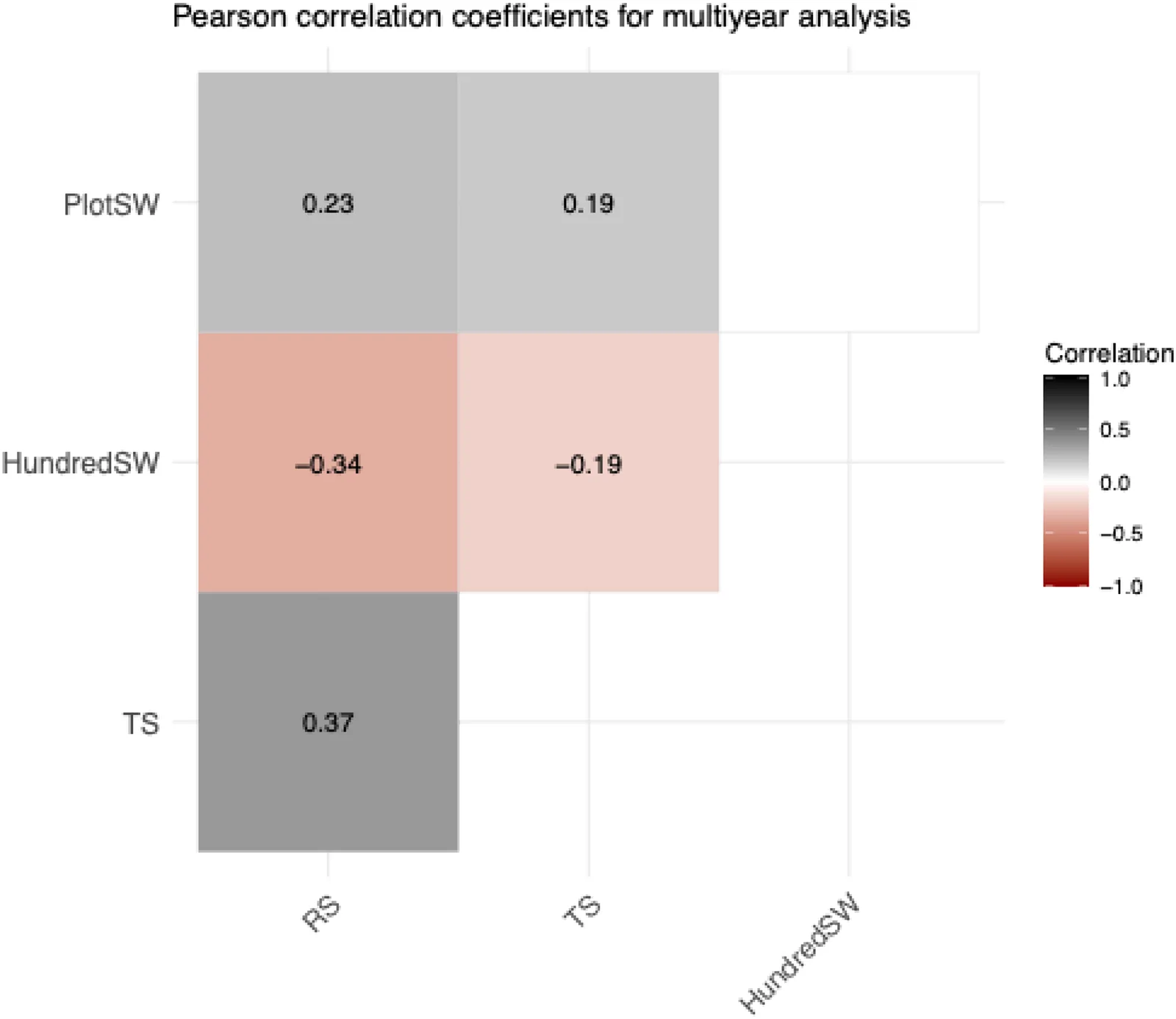
Correlation matrix of resistant starch (RS), total starch (TS), 100-seed weight (Hundred-SW), and Plot-seed weight (Plot-SW) in lentils multiyear environment. The color scale represents the strength and direction of the correlation: black indicates positive correlation, red indicates negative correlation, and white indicates no correlation. Values shown are Pearson correlation coefficients. Only statistically significant correlations (p ≤ 0.05) are colored and labeled; non-significant correlations are left blank.

### 3.3 Marker traits associations

A genome-wide association study (GWAS) conducted using the BLINK model identified nine SNPs significantly associated with RS concentration in a diverse lentil panel grown in three environments, Irwell, Spillman, and Central Ferry, as well as in a multi-environment BLUPs-based analysis. The identified SNPs were distributed across chromosomes 1, 2, 4, 5, and 7, and explained 9.1% to 38.5% of the phenotypic variance. In Irwell, one SNP (CHR4_389689669) on chromosome 4 explained 24.7% of the variance. In Spillman, two SNPs on chromosome 4 (CHR4_6631374 and CHR4_349859085) were associated with RS and explained 25.1% and 14.8% of the variance, respectively. In Central Ferry, four significant SNPs were detected on chromosomes 1, 5, and 7, with CHR5_145398145 accounting for the highest variance (26.9%), followed by CHR1_397487653 (13.7%), CHR5_472805423 (10.7%), and CHR7_509092321 (9.1%). In the multi-environment BLUPs-based analysis, two SNPs, CHR1_513323908 on chromosome 1 explained 11.4% of the phenotypic variance and CHR2_24807231 on chromosome 2, had the strongest association of all SNPs and accounted for 38.5% of the phenotypic variance (**Figure 4**; **Table 2**).

**Figure 4:**
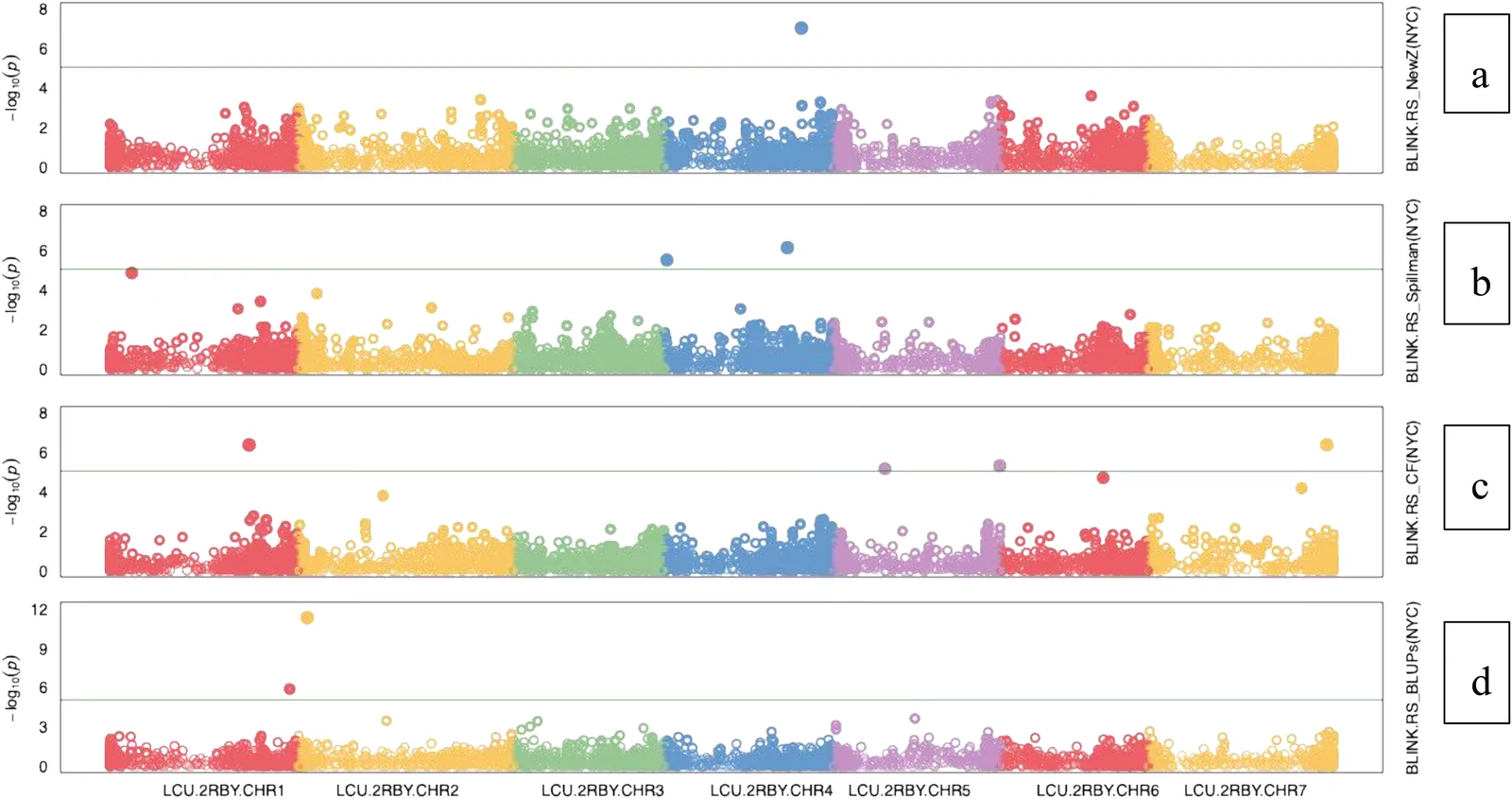
Manhattan plots show significant SNP associations for resistant starch (RS) concentration in lentils across multiple environments. **(a)** RS in Irwell, NZ; **(b)** RS in Spillman, WA; **(c)** RS in Central Ferry, WA; **(d)** RS using BLUPs across the three environments. The green horizontal line indicates the significance threshold.

**Table 2:** Significant MTAs associated with resistant Starch (RS), total Starch (TS), 100-seed weight (100-SW), and plot seed weight (Plot-SW) in lentils identified across multiple environments.

Environment	Trait	MTAs	Chr	Pos	P-value	MAF	Effect	PVE (%)
Irwell	RS	CHR4_389689669	4	389689669	9.3094E-08	0.31	1.84	24.66
Spillman	RS	CHR4_6631374	4	6631374	3.0698E-06	0.19	-1.68	25.11
Spillman	RS	CHR4_349859085	4	349859085	7.299E-07	0.18	-1.57	14.81
Central Ferry	RS	CHR1_397487653	1	397487653	4.226E-07	0.11	-1.55	13.74
Central Ferry	RS	CHR5_145398145	5	145398145	6.714E-06	0.49	1.07	26.94
Central Ferry	RS	CHR5_472805423	5	472805423	4.6824E-06	0.41	-1.00	10.68
Central Ferry	RS	CHR7_509092321	7	509092321	4.0788E-07	0.25	-1.23	9.07
Multi-year	RS	CHR1_513323908	1	513323908	8.1707E-07	0.23	-1.36	11.35
Multi-year	RS	CHR2_24807231	2	24807231	1.2044E-09	0.10	2.02	38.50
Spillman	TS	CHR2_64012776	2	64012776	7.3144E-08	0.06	-2.20	42.60
Spillman	TS	CHR5_145398145	5	145398145	3.5472E-07	0.49	1.16	13.71
Central Ferry	TS	CHR3_327084734	3	327084734	1.732E-06	0.34	-1.18	20.87
Irwell	100-SW	CHR1_319564320	1	319564320	2.6613E-07	0.34	-0.25	26.41
Irwell	100-SW	CHR5_273800854	5	273800854	9.293E-07	0.24	-0.56	31.45
Spillman	100-SW	CHR2_531141625	2	531141625	3.5711E-06	0.44	0.20	2.87
Spillman	100-SW	CHR5_273800854	5	273800854	2.6207E-08	0.24	-0.49	27.41
Spillman	100-SW	CHR5_428451688	5	428451688	7.4247E-07	0.16	0.28	17.02
Spillman	100-SW	CHR7_486711048	7	486711048	6.8874E-06	0.46	-0.17	12.03
Central Ferry	100-SW	CHR1_319564320	1	319564320	1.2837E-16	0.35	-0.31	16.52
Central Ferry	100-SW	CHR2_604760130	2	604760130	4.74E-08	0.16	-0.23	3.96
Central Ferry	100-SW	CHR3_343703728	3	343703728	2.9862E-07	0.13	0.24	10.74
Central Ferry	100-SW	CHR5_273800854	5	273800854	8.8332E-14	0.24	-0.70	20.09
Central Ferry	100-SW	CHR5_434044254	5	434044254	2.1342E-08	0.26	0.18	4.66
Central Ferry	100-SW	CHR6_134836269	6	134836269	1.3969E-12	0.34	-0.21	14.36
Central Ferry	100-SW	CHR7_503203241	7	503203241	3.2077E-07	0.19	0.21	7.69
Multi-year	100-SW	CHR1_319564320	1	319564320	1.3824E-23	0.35	-0.45	17.64
Multi-year	100-SW	CHR2_66839705	2	66839705	2.2665E-15	0.11	0.56	32.35
Multi-year	100-SW	CHR2_604760130	2	604760130	9.4446E-12	0.16	-0.33	10.24
Multi-year	100-SW	CHR3_1409547	3	1409547	6.475E-09	0.07	0.33	8.50
Multi-year	100-SW	CHR4_472773527	4	472773527	1.5411E-10	0.27	0.26	6.71
Multi-year	100-SW	CHR5_115055606	5	115055606	2.2689E-07	0.34	-0.27	5.11
Multi-year	100-SW	CHR7_476056054	7	476056054	4.4525E-09	0.41	-0.26	7.19
Irwell	Plot-SW	CHR2_318713372	2	318713372	8.8075E-07	0.18	-32.63	15.80
Irwell	Plot-SW	CHR4_416704306	4	416704306	3.8333E-09	0.48	52.43	26.75
Spillman	Plot-SW	CHR2_523267693	2	523267693	3.5253E-07	0.13	-19.55	14.16
Spillman	Plot-SW	CHR7_9957088	7	9957088	2.4666E-09	0.26	21.52	20.42
Central Ferry	Plot-SW	CHR2_36120465	2	36120465	6.2391E-09	0.19	-22.21	25.72
Multi-year	Plot-SW	CHR2_207072870	2	207072870	1.8472E-07	0.19	27.74	14.39
Multi-year	Plot-SW	CHR2_612835328	2	612835328	5.488E-07	0.14	22.50	14.73
Multi-year	Plot-SW	CHR3_47177231	3	47177231	1.7731E-07	0.29	25.14	10.35
Multi-year	Plot-SW	CHR3_246147336	3	246147336	1.0814E-06	0.46	-17.37	10.12
Multi-year	Plot-SW	CHR6_41537280	6	41537280	2.0136E-06	0.38	-15.36	10.67
Multi-year	Plot-SW	CHR6_403741802	6	403741802	6.9176E-09	0.20	-23.22	11.52

The table provides information on environment, trait, MTA name, chromosome (Chr), physical position (Pos), p-value, minor allele frequency (MAF), allelic effect, and phenotypic variance explained (PVE%) for each association.

For total starch concentration, no significant MTAs were detected using the across-environment genotype BLUPs. However, three environment-specific MTAs were identified, including two at Spillman and one at Central Ferry. In Spillman, CHR2_64012776 on chromosome 2 had the strongest association, with a minor allele frequency (MAF) of 0.057 and explained 42.6% of the phenotypic variance. Another SNP detected in Spillman, CHR5_145398145, had a MAF of 0.49 and contributed 13.7% to the variance. In the Central Ferry environment, one SNP, CHR3_327084734, located on chromosome 3, was associated with total starch, with a MAF of 0.34 and explaining 20.9% of the phenotypic variance (**Figure 5**; **Table 2**).

**Figure 5:**
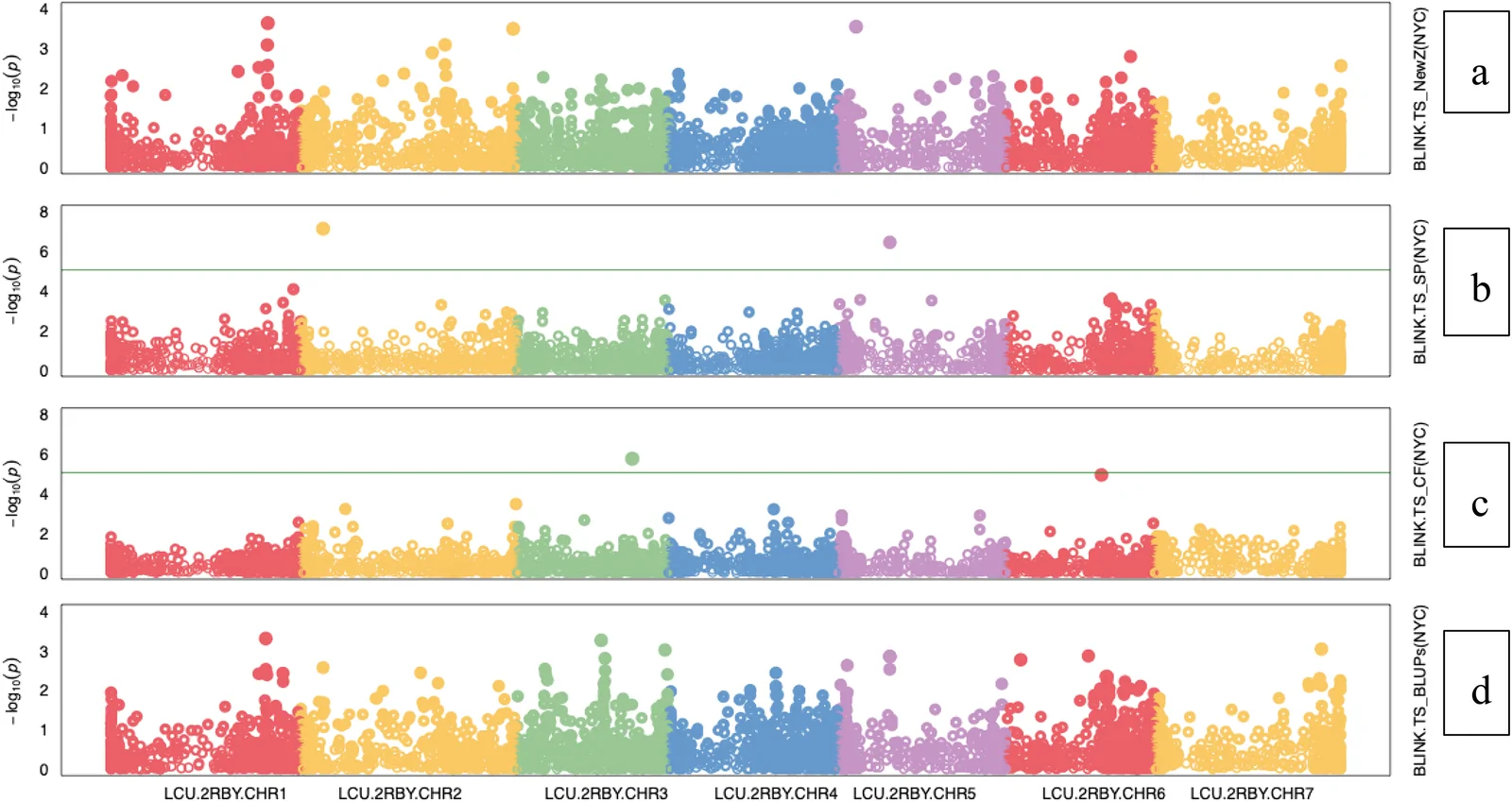
Manhattan plots showing significant SNP associations for total starch (TS) concentration in lentils across multiple environments. **(a)** TS in Irwell, NZ; **(b)** TS in Spillman, WA; **(c)** TS in Central Ferry, WA; **(d)** TS using BLUPs across the three environments. The green horizontal line indicates the significance threshold.

For 100-SW, a total of 20 significant SNPs were identified across Irwell, Spillman, Central Ferry, and multi-year (BLUPs-based) analyses. In Irwell, two SNPs were detected on chromosomes 1 and 5, explaining 26.4% and 31.5% of the phenotypic variance, respectively. In Spillman, five SNPs were identified on chromosomes 2, 5, and 7, with PVE values ranging from 2.9% to 27.4%. In Central Ferry, eight SNPs were detected on chromosomes 1, 2, 3, 5, 6, and 7, explaining between 3.9% and 20.1% of the phenotypic variance. In the multi-year analysis, six SNPs were associated with 100-SW on chromosomes 1, 2, 3, 4, 5, and 7, with PVE values ranging from 5.1% to 32.4%. Two SNPs, CHR1_319564320 and CHR5_273800854, were identified in three different environments (**Figure 6**; **Table 2**).

**Figure 6:**
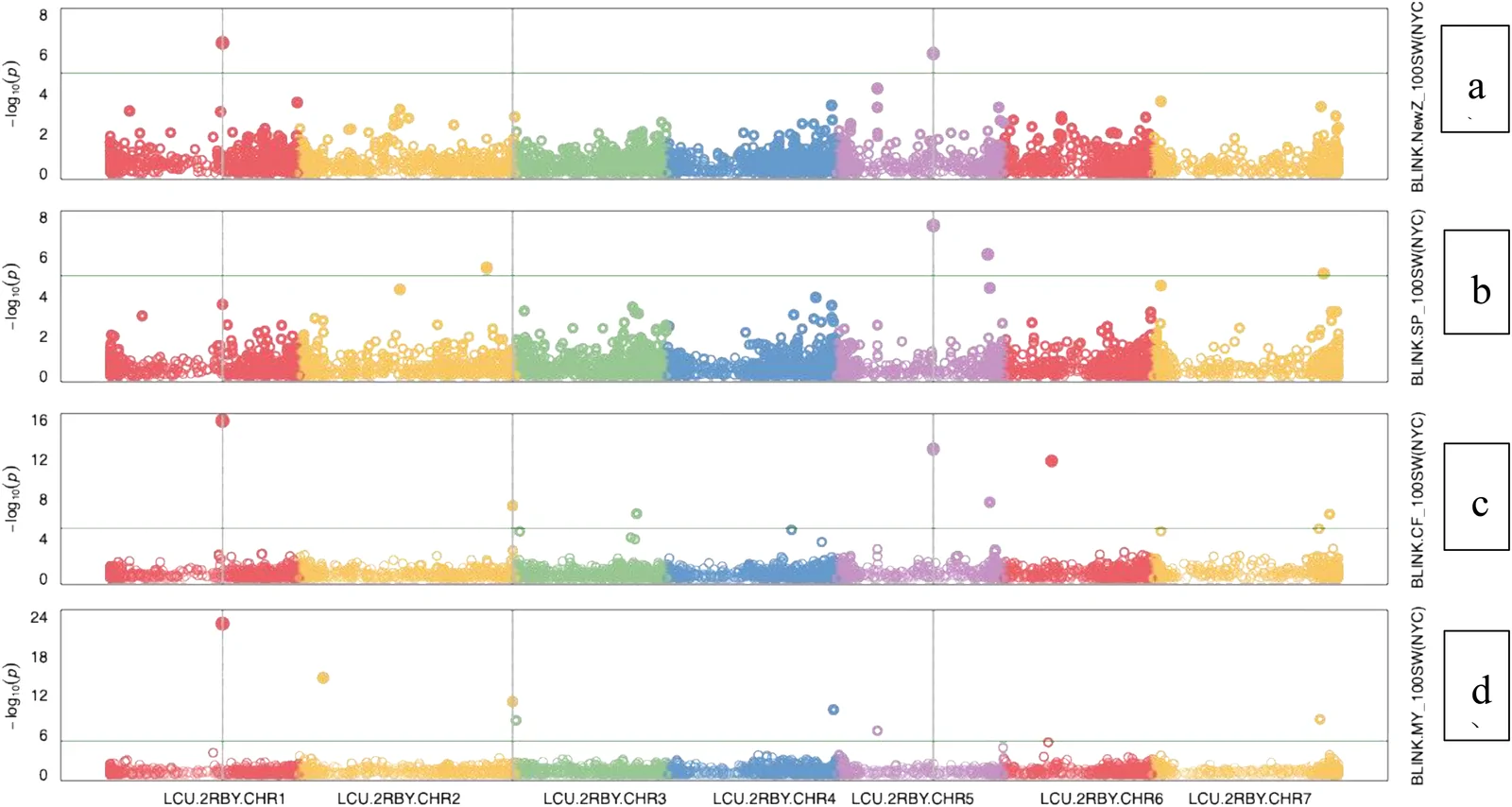
Manhattan plots showing significant SNP associations for 100-seed weight (100-SW) in lentils across multiple environments. Panels represent **(a)** Irwell, NZ; **(b)** Spillman, WA; **(c)** Central Ferry, WA; and **(d)** multi-year analysis based on the average across environments. The green horizontal line indicates the genome-wide significance threshold. Gray vertical lines highlight SNPs that are commonly associated across multiple environments.

For plot seed weight, a total of 11 significant SNPs were identified across Irwell, Spillman, Central Ferry, and multi-year analyses. In Irwell, two SNPs were detected on chromosomes 2 and 4, explaining 15.8% and 26.8% of the phenotypic variance, respectively. In Spillman, two SNPs were identified on chromosomes 2 and 7, with phenotypic variance explained (PVE) values of 14.2% and 20.4%. One SNP was detected in the Central Ferry environment on chromosome 2, explaining 25.7% of the variance. In the multi-year analysis, six SNPs were identified across chromosomes 2, 3, and 6, with PVE values ranging from 10.1% to 14.7% (**Figure 7**; **Table 2**).

**Figure 7:**
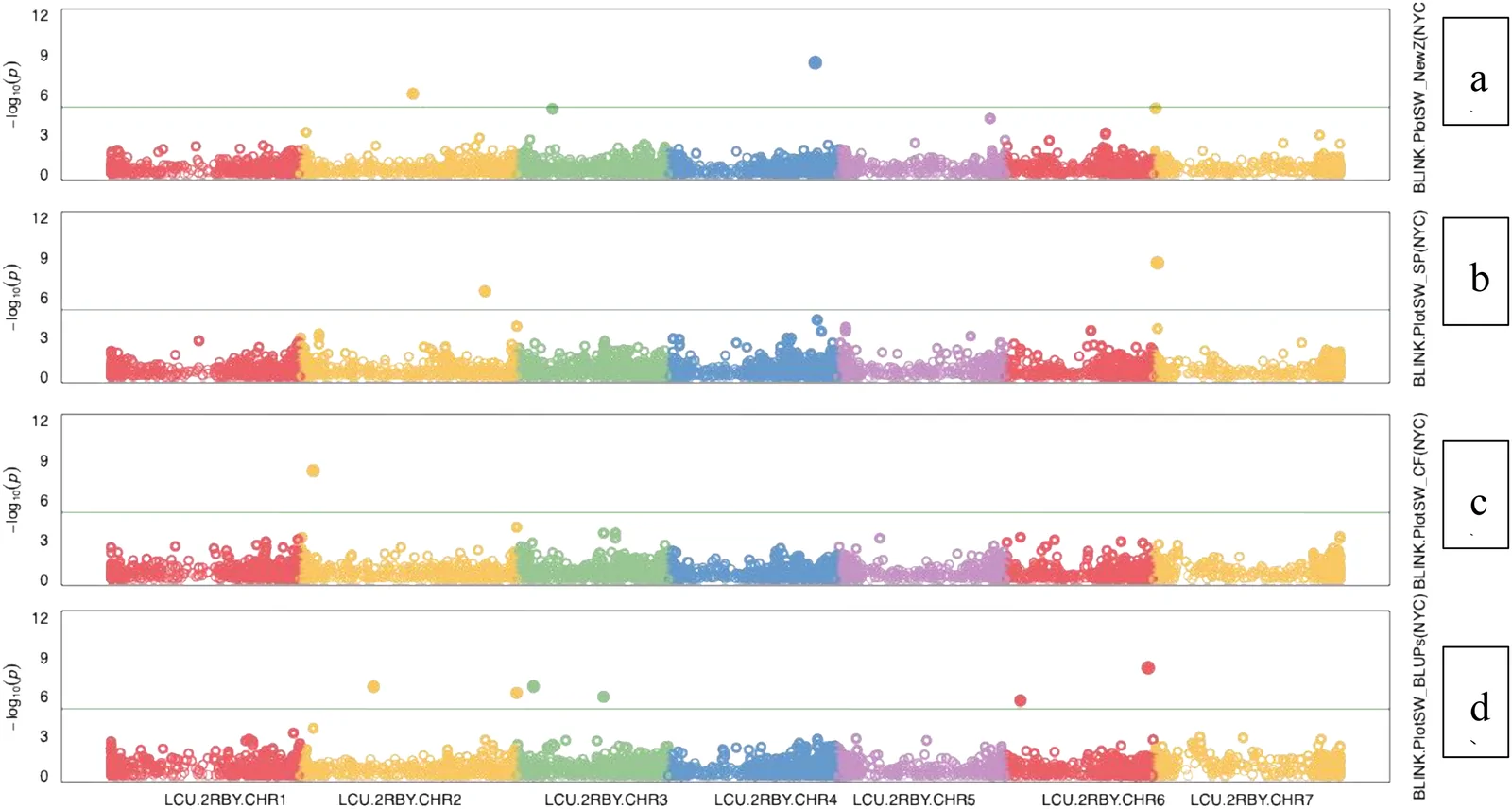
Manhattan plots show significant SNP associations for plot seed weight (Plot-SW) in lentils across multiple environments. Panels represent **(a)** Irwell, NZ; **(b)** Spillman, WA; **(c)** Central Ferry, WA; and **(d)** multi-year analysis based on the average across environments. The green horizontal line indicates the genome-wide significance threshold.

### 3.4 Putative candidate genes and functional annotation

Putative candidate genes surrounding the significant RS-associated MTAs were identified using the *Lens culinaris* CDC Redberry v2.0 reference genome and functionally annotated based on their *Arabidopsis thaliana* homologs (**Supplementary Table 2**). Functional enrichment analysis revealed significant representation of several biological processes and molecular functions. Notably, genes associated with starch metabolism were identified, including homologs of starch synthases (*SS1*, *SS2*, *SS3*, and *SS4*) and granule-bound starch synthase 1 (*GBSS1*). Consistent with these annotations, GO molecular function analysis showed significant enrichment for alpha-1,4-glucan glucosyltransferase activity and glucosyltransferase activity, while GO cellular component analysis identified significant enrichment for the amyloplast (**Figures 8**, **9**).

**Figure 8:**
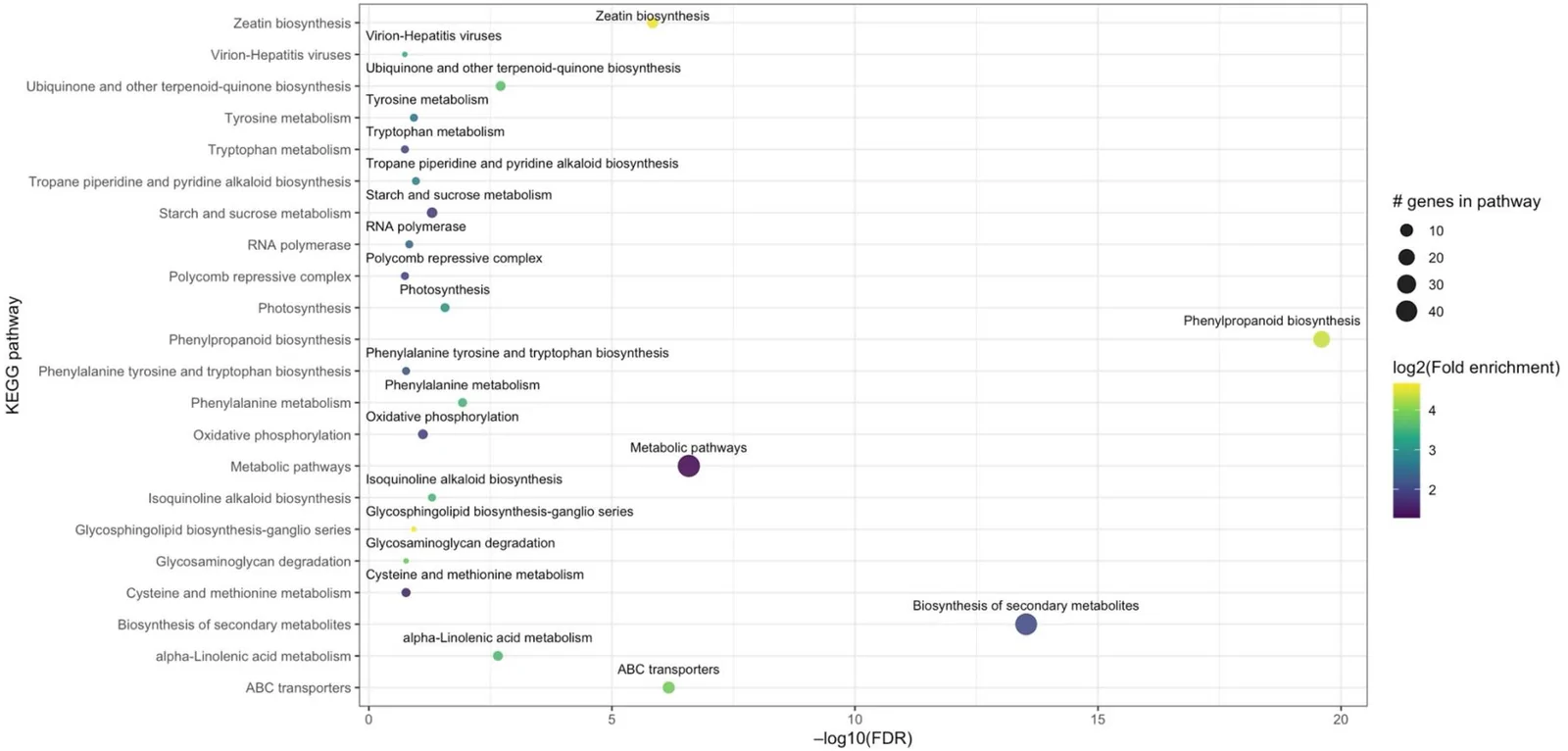
KEGG pathway enrichment of candidate genes associated with resistant starch in lentils. Significantly enriched KEGG pathways (FDR< 0.05) identified from candidate genes within 100-kb LD windows of RS-associated MTAs are displayed. The x-axis shows pathway significance as –log_10_(FDR). Bubble size corresponds to the number of genes mapped to each pathway, while bubble color indicates log_2_(fold enrichment), reflecting the degree of over-representation relative to the background gene set. Labels denote pathway names.

**Figure 9:**
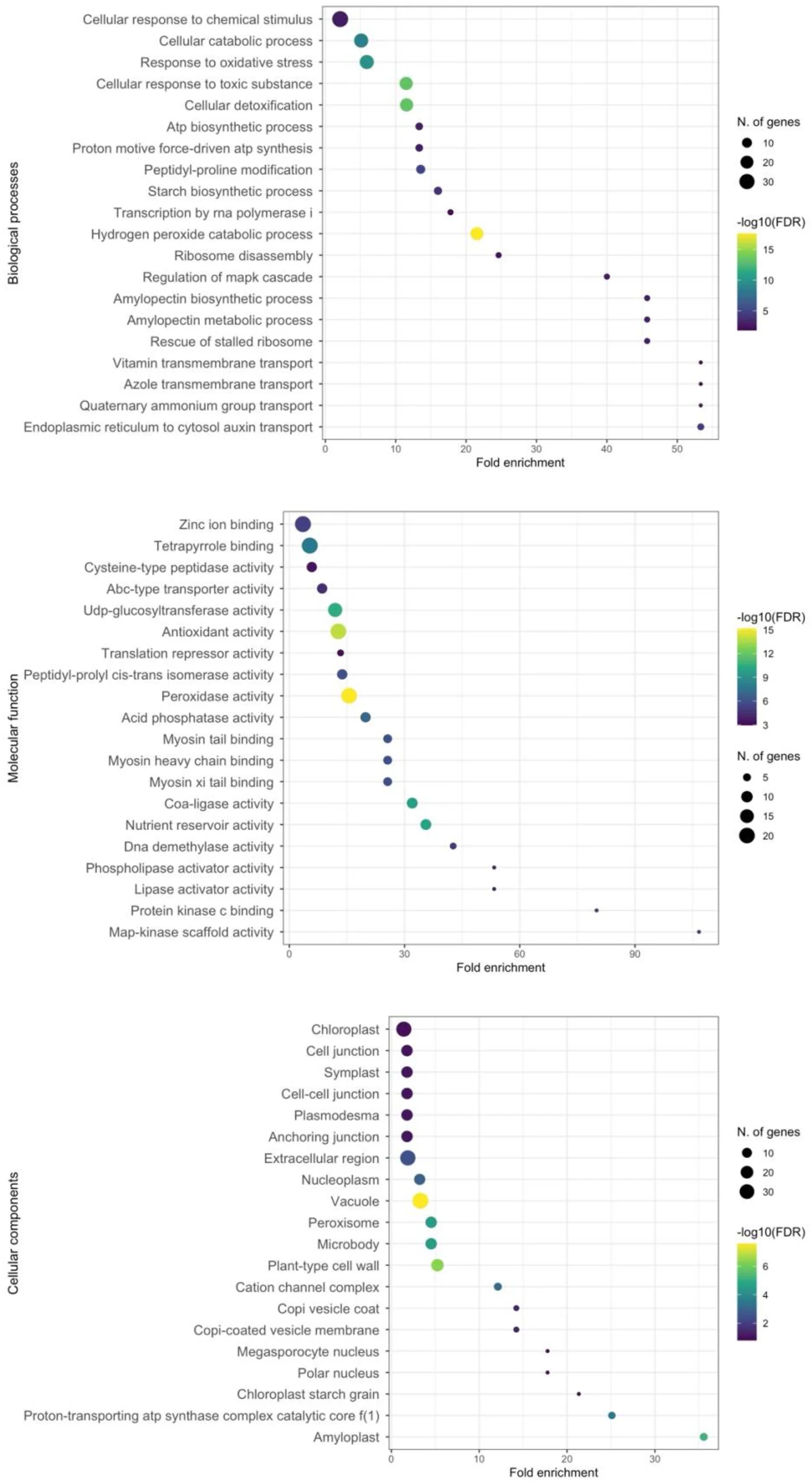
Gene ontology (GO) enrichment analysis of candidate genes associated with resistant starch in lentils. Significantly enriched GO terms (FDR< 0.05) identified from candidate genes within 100-kb LD windows of RS-associated MTAs are shown for biological process (top panel), molecular function (middle panel), and cellular component (bottom panel). The x-axis represents fold enrichment, bubble size indicates the number of genes assigned to each GO term, and bubble color corresponds to –log_10_(FDR), reflecting statistical significance. Redundant GO terms were reduced using semantic similarity analysis prior to visualization.

KEGG enrichment analysis also identified several significantly enriched pathways, including phenylpropanoid biosynthesis, biosynthesis of secondary metabolites, metabolic pathways, ABC transporters, and zeatin biosynthesis. Additional enriched GO categories were associated with hydrogen peroxide metabolism, cellular detoxification, reactive oxygen species metabolism, peroxidase activity, and antioxidant activity. Together, these results identified candidate genes and functional categories potentially associated with variation in resistant starch and related seed traits (**Figures 8**, **9**).

Functional annotation of the significant MTAs further showed that the identified variants occurred in coding and noncoding genomic regions. Among the 37 unique, significant MTAs identified across the evaluated traits, 15 were located within coding sequences, including 10 synonymous variants, four nonsynonymous variants, and one stop-gained variant. Fourteen MTAs were intronic, two were located in 3′ UTRs, and six were intergenic. Among the RS-associated MTAs, CHR4_6631374 was a nonsynonymous variant predicted to result in a Val-to-Glu substitution, whereas CHR4_389689669 and CHR7_509092321 were synonymous variants (**Supplementary Table 2**).

## 4 Discussion

Resistant starch is a functionally important carbohydrate that modulates postprandial glucose and insulin responses and has been associated with improved insulin sensitivity, traits relevant to dietary quality and human health (Ashwar et al., 2016; Robertson et al., 2005; Maki et al., 2012; Birt et al., 2013; Dong et al., 2025). Controlled dietary interventions have demonstrated that increased RS intake reduces postprandial glycemia and enhances whole-body insulin action in overweight adults (Maki et al., 2012; Bodinham et al., 2014). An eight-week supplementation study providing 40 g/day RS further reported reductions in body weight (~2.8 kg) and improvements in insulin resistance, suggesting that RS content in staple foods may contribute to favorable metabolic outcomes (Li et al., 2024). In addition to these systemic effects, RS functions as a prebiotic substrate for gut microbiota, with microbial fermentation producing short-chain fatty acids (SCFAs), particularly butyrate, that support gut epithelial integrity and exert anti-inflammatory effects, thereby linking RS composition to broader physiological processes (Peng et al., 2009; Berni Canani et al., 2012; Koh et al., 2016; Baptista et al., 2024; Chen et al., 2024). RS enriches key degraders such as *Ruminococcus bromii* and downstream butyrate producers, shifting the microbiome toward a more butyrogenic profile with systemic metabolic payoffs (Ze et al., 2012; Louis and Flint, 2017). These SCFA-mediated pathways link the gut to host glucose and lipid metabolism, offering a biologically plausible explanation for the metabolic benefits observed in human studies (den Besten et al., 2013; Koh et al., 2016). The two primary mechanisms of RS, dampening glycemic load in the small intestine and feeding butyrate-producing microbes in the colon, provide a clear, mechanism-based rationale for prioritizing RS in staple foods, like lentils, when the goal is long-term metabolic and gut health (Ze et al., 2012; Birt et al., 2013; Koh et al., 2016; Louis and Flint, 2017).

Across the three environments, RS showed wide, site-dependent variation (range 8.4%–23.6% at Central Ferry, 8.5%–28% at Spillman, and 7.3%–27.8% at Irwell), whereas TS varied more narrowly (31.1%–47.1% at Central Ferry, 34.2%–53.8% at Spillman, and 31.6%–49.8% at Irwell), a pattern consistent with literature indicating that starch quantity in lentil is relatively stable while resistance to digestion is more sensitive to environmental and processing effects on granule structure and retrogradation dynamics (Aguilera et al., 2009; Ninou et al., 2019; Nahar et al., 2024). Reported carbohydrate/starch levels for lentils are approximately 40%–45% of seed weight (Ninou et al., 2019; Canadian Grain Commission, 2020a, b), aligning with the TS means observed in this study, while RS fractions are known to shift with growth conditions and post-harvest applications (Siva et al., 2020). The stronger environmental signal on RS is also compatible with broader pulse data showing modest RS in cooked pulses (~6% of dry matter) that can change with processing and cooling, underscoring why genotype-by-environment (G×E) and post-processing interactions need to be considered when ranking accessions for RS (Nahar et al., 2024). The phenotypic distributions reinforce these conclusions and suggest practical selection levers. For RS, the highest median and broader interquartile range were observed at Spillman, suggesting that this environment supports greater expression of high-RS genotypes, with several potential outliers above 25%. Total starch showed relatively similar medians across environments, though Irwell displayed slightly higher variability, while Central Ferry exhibited a tighter distribution, possibly reflecting environmental constraints on starch accumulation. For 100-SW, Irwell exhibited the highest median and the widest range of values, followed by Central Ferry, while Spillman showed the lowest median and a narrower distribution (**Figure 1**). These patterns suggest both genetic and environmental influences on seed size. The wide variation observed, particularly in Irwell, aligns with previous studies reporting broad diversity and deviations from normality in seed size within lentil germplasm panels (Fedoruk et al., 2013; Verma et al., 2015). Plot-SW was markedly higher and more variable at Irwell, while Central Ferry had the lowest median and narrowest spread, consistent with environmental limitations on yield (Shrestha et al., 2006; Delahunty et al., 2018; Tziouvalekas et al., 2022). These results highlight the importance of testing genotypes across diverse environments to identify lines with desirable combinations of RS, TS, and yield traits under variable growing conditions.

Lower RS was correlated with orange/pink seed color percentage, and higher RS concentration was positively correlated with brown/black seed color (**Figure 2**). This agrees with what is known about lentil seed coats: darker seed coats accumulate more phenolics (especially proanthocyanidins) that hinder enzymatic access to starch granules and slow hydrolysis, which can elevate the resistant fraction, whereas lighter seed coats generally carry less of these inhibitors (Dueñas et al., 2003; Mirali et al., 2017; Duijsens et al., 2023). Strong positive correlations among color traits are also expected because seed‐coat colors co-vary with phenylpropanoid/flavonoid pathway outputs that are coordinated genetically, so color classes often cluster together across environments (Mirali et al., 2017; Mishra et al., 2022). Processing studies underscore the mechanism: keeping the hull (rich in fiber and polyphenols) or maintaining polyphenol–starch complexes reduce starch digestibility, while splitting/dehulling breaks these barriers and reduces RS supporting the direction of correlations (Eyaru et al., 2009; Balwinder Singh et al., 2017; Duijsens et al., 2023). The consistency across environments aligns with evidence that while environment can shift absolute phenolic levels, genotype/color-class differences are stable enough to produce similar correlation structures across sites (Mirali et al., 2017). The positive correlation between RS and TS (**Figure 3**) is consistent with evidence that RS increases with aspects of starch architecture, particularly higher amylose content and retrogradation, features common in legume starches that are generally less digestible than cereal starches (Yadav et al., 2010; Bajaj et al., 2018; Baptista et al., 2024). The negative correlation between RS and 100-SW aligns with reports that 1000-seed weight in lentil is negatively correlated with amylose concentration, a principal driver of RS formation (Tahir et al., 2011), and with observations that RS expression and yield components are sensitive to environment and management (Yadav et al., 2010; Bajaj et al., 2018; van Eeuwijk et al., 2019; Chen et al., 2022). The weak negative association between 100-SW and TS suggests that composition size relationships can be panel- and environment-dependent; notably, other lentil studies have found positive correlations between 1000-seed weight and starch concentration, underscoring genetic and environmental plasticity in these covariances (Tahir et al., 2011). RS showed moderate-to-high generalized broad-sense heritability (H² = 0.632), indicating that genetic differences among genotypes contributed substantially to RS variation in this population. In contrast, TS showed low heritability (H² = 0.182), consistent with the limited overall genotypic variation observed for this trait. From a breeding perspective, the moderate-to-high heritability of RS, together with the modest absolute values of these correlations, suggests potential for selection for increased RS while considering correlated seed traits and validating performance across environments.

The GBS approach used in this study identified a sufficient number of high-quality SNPs to enable GWAS analyses. The BLINK model yielded well-fitted *Q*–*Q* plots, indicating a good fit of test statistics and appropriate control of false positives during the identification of MTAs. The detection of MTAs for RS, TS, 100-SW, and Plot-SW provides key findings into the genetic control of seed nutritional quality and yield traits in lentils. A total of 43 significant MTAs associated with these traits were identified across all seven lentil chromosomes (**Figures 4**–**7**; **Table 2**), reaffirming the polygenic architecture underlying these complex traits (Fedoruk et al., 2013; Verma et al., 2015; Johnson et al., 2021; Rajendran et al., 2021; Padhy et al., 2025).

Notably, the MTA CHR2_24807231 demonstrated the strongest association, explaining 38.5% of phenotypic variance in the multi-environment BLUPs-based model. The MTAs CHR4_389689669 and CHR4_349859085 detected on Chr 4 in Irwell and Spillman environments could be further characterized through haplotype analysis or fine mapping to clarify their genetic effects. Previous work in lentils by Chen et al. (2022) demonstrated significant environmental and genotypic effects on starch concentration, reinforcing the importance of multi-environment evaluations when targeting starch-related traits. In the present study, no MTAs for RS overlapped with those reported by Johnson et al. (2021), which is expected given substantial differences in population, growing environments, and analytical approaches in this study. Given that lentil exhibits long blocks of linkage disequilibrium (Guerra‐Garcia et al., 2022), distinct lead MTAs may still represent the same broad genomic region, while contrasting environments may uncover different loci associated with RS. To refine these associations, windows of ±300–500 kb should be examined for potential colocalization, and the most impactful loci, such as the strong signal on Chr 2, can be validated through KASP genotyping across shared germplasm and environments. Most MTAs identified for RS were specific to individual environments, while two associations were detected using across-environment BLUPs. Although RS showed moderate-to-high broad-sense heritability, the effects and detectability of individual loci may vary among growing environments. Consequently, MTAs detected in only one environment should be considered putative environment-specific associations rather than stable loci. Validation of these associations across additional years, environments, and independent genetic populations will be necessary to determine their stability and potential utility in marker-assisted breeding. In contrast to RS, TS concentration appeared to be influenced by fewer but more impactful loci, particularly in the Spillman environment where CHR2_64012776 accounted for 42.6% of the phenotypic variance. This pattern suggests that a limited number of strong-effect loci may operate under specific conditions. Large-effect associations for seed carbohydrate composition have also been reported in two legume single environment studies; for example, a lentil GWAS on prebiotic carbohydrates, including resistant starch, identified significant MTAs with sizable effects (Johnson et al., 2021), and a chickpea GWAS detected major signals for raffinose family oligosaccharides (RFOs) such as raffinose and stachyose (Elango et al., 2022). However, single-environment GWASs suffer from limited reproducibility across environments due to environmental effects, low sample size, and population–structure confounding (Korte and Farlow, 2013). Recent approaches that integrate multi-environment trials and meta-analytic GWAS (De Walsche et al., 2025) demonstrate increased robustness and the ability to detect both stable and environment-dependent loci, highlighting the added value of multi-environment designs for baseline nutritional traits such as resistant starch. Multi-environment GWASs in chickpea have identified significant MTAs for dietary fiber, explaining up to 38.6% of the phenotypic variance (Sari et al., 2024b). In pea, classical mutations at the starch-branching enzyme loci (*r*, *r_b_*) are known to drastically alter seed starch composition, illustrating that single genes can exert major effects on carbohydrate traits (Yu et al., 2021). Notably, the CHR5_145398145 was identified in association with both RS and TS. This co-localization may reflect shared regulatory mechanisms affecting starch biosynthesis and distribution, particularly when RS makes up a large portion of the total starch concentration.

A total of 20 significant MTAs associated with 100-SW were detected, including CHR1_319564320 and CHR5_273800854, which were consistently identified across three or four environments. This level of consistency is especially valuable for breeding. It aligns with earlier lentil studies where QTL for seed size or 100-seed weight have shown stability; for example, Fedoruk et al. (2013) reported multiple stable QTL for seed weight and seed dimensions in several linkage groups, and Verma et al. (2015) mapped QTL for seed weight and seed size in lentil on LG4 with large PVE. More recently, Padhy et al. (2025) combined QTL mapping, GWAS, and transcriptomics to reveal molecular signatures controlling seed size/weight, highlighting promising candidate genes. For Plot-SW, 11 significant MTAs were found, with moderate PVE values (≈10%–26.8%), as is typical given its sensitivity to environmental variation. Two MTAs on chromosome 2, CHR2_36120465 and CHR2_207072870, contributed significantly in both single-environment and multi-year models. The detection of environment-specific loci again underlines the importance of genotype by environment interaction in lentil yield traits (Verma et al., 2015; Rajendran et al., 2021; Padhy et al., 2025).

To explore potential mechanisms underlying the marker–trait associations (MTAs) identified in this study, candidate genes located within a 100-kb window of the nine significant MTAs for resistant starch were examined using KEGG and gene ontology (GO) enrichment analyses (**Figures 8**, **9**). These genes were significantly enriched in pathways directly related to starch metabolism, including starch and sucrose metabolism, amylopectin biosynthetic and metabolic processes (**Figure 8**), and ATP biosynthesis, highlighting core carbohydrate biosynthesis and energy metabolism as central drivers of RS variation. Strong enrichment was also observed for phenylpropanoid biosynthesis, biosynthesis of secondary metabolites, and ABC transporters, suggesting additional roles for cell wall modification, metabolite transport, and stress-related metabolic processes in shaping RS accumulation. GO biological process enrichment further revealed overrepresentation of oxidative stress response, cellular detoxification, and hydrogen peroxide catabolic processes, indicating that redox regulation and stress signaling may indirectly influence starch biosynthesis (Santelia et al., 2015; Skryhan et al., 2018). At the molecular function level, enrichment of UDP-glycosyltransferase activity, β-galactosidase activity, peroxidase activity, and starch synthase related functions support coordinated regulation of carbohydrate modification and cell wall remodeling. Cellular component enrichment localized many of these proteins to the amyloplast, chloroplast, vacuole, and cell wall, consistent with known sites of starch synthesis and storage. Genes with putative roles in RS biosynthesis or regulation are discussed below in detail. A comprehensive list of all 55 putative candidate genes identified is provided in **Supplementary Table 2**.

The strongest association, MTA CHR2_24807231 on Chr 2, was located approximately 67.6 kb from *Lcu.2RBY.2g009830* (**Figure 8**; **Supplementary Table 2**), a candidate gene orthologous to *Arabidopsis thaliana SS3* (soluble starch synthase III) (**Figure 8**). *SS3* is involved in elongating intermediate amylopectin chains and influences starch granule structure (Fujita et al., 2007; Seung et al., 2015). These structural features may be relevant to RS3 formation, the predominant form of resistant starch in lentils, which occurs through retrogradation of amylose-rich starch upon cooling (Chen et al., 2017; Xu et al., 2020; Bangar et al., 2022). Recent studies in other species provide additional biological support for SS3 as a candidate. In rice, mutations in *SSIIIa* have been associated with increased RS, including in combination with other starch biosynthesis genes (Pinson et al., 2025; Wang et al., 2025). However, the evidence in the present study is based on the genomic proximity of *SS3* and resistant starch was experimentally evaluated. Therefore, *SS3* should be considered a putative candidate gene for lentil resistant starch, and further studies, including gene expression and functional validation, are required to determine its role in RS accumulation.

The MTA CHR5_472805423 on Chr 5 was located 94 kb and 96.4 kb from *Lcu.2RBY.5g073830* and *Lcu.2RBY.5g073840*, respectively, both of which were annotated as *BGAL*s (**Figures 8**, **9**). These β-galactosidases (*BGAL*s) cleave galactosyl residues from pectins and hemicelluloses in plant cell walls, potentially altering porosity and mechanical resistance (Carey et al., 2001; Gantulga et al., 2008). In legumes, starch granules are often encapsulated within intact cotyledon cells or cell walls (Dhital et al., 2016; Bhattarai et al., 2017), which can physically limit enzymatic access and contribute to RS1 formation (Englyst et al., 1992; Kraithong et al., 2022). Variation in *BGAL* activity may therefore influence RS by modulating cell wall loosening and starch bio-accessibility, offering a pathway distinct from, but complementary to, *SS3* for enhancing resistant starch concentration. However, the present study measured total RS and did not quantify cell wall components or distinguish among RS subtypes. Therefore, a specific relationship between *BGAL* and RS1 cannot be established from the present results and requires further investigation.

Finally, the MTA CHR4_389689669 co-localized with a tandem cluster of UDP-glycosyltransferase (*UGT*) genes (**Figures 8**, **9**), including UGT85A23 and UGT85A24 (*Lcu.2RBY.4g060560*–*580*). *UGT*s are involved in the glycosylation of diverse small molecules, and emerging evidence links their activity to sugar signaling and metabolic partitioning during seed development (Bowles et al., 2006; Yang et al., 2023). Although their direct role in starch biosynthesis or resistant starch formation is not fully understood, these *UGT*s may act as upstream regulators influencing carbohydrate allocation or developmental processes that indirectly affect RS accumulation.

In conclusion, this study provides new insights into the genetic architecture of RS and related seed traits in lentil by integrating multi-environment phenotyping, mixed-model analysis, GWAS, candidate gene identification, and functional enrichment analyses. The observed relationships between RS, seed coat color, and seed weight are consistent with previous reports linking polyphenol accumulation and starch biosynthesis to seed composition and functionality. Mixed-model analysis demonstrated significant genetic variation for RS, while total TS showed limited overall genetic variation across environments. GWAS identified nine significant MTAs for RS, including a major locus on chromosome 2 located near the *SS3* gene, which plays an important role in amylopectin chain elongation and resistant starch formation. Additional MTAs were located near genes involved in cell wall remodeling (*BGALs*) and sugar metabolism (*UGTs*), suggesting potential roles for carbohydrate metabolism and cell wall related processes RS variation. However, these candidate genes were identified based on genomic proximity and functional annotation and have not been functionally validated in lentil. Collectively, these findings highlight genomic regions and putative candidate genes potentially associated resistant starch and provide a basis for further investigation of the genetic control of this trait. Future research should focus on converting these SNPs into breeder-friendly KASP assays and validating their associations in independent lentil populations. Once validated, these markers could facilitate marker-assisted selection for the development of nutritionally enhanced lentil cultivars.

## Data Availability

All data generated or analyzed during this study are publicly available. The datasets are included within this article and its **Supporting Information** or are accessible through the following repositories: Ma et al. (2020), **Supplementary Materials** (https://www.mdpi.com/1422-0067/21/6/2129/s1) and the Pulse Crop Database (PulseDB), Genomic, Genetic, and Breeding Resources for Pulse Crop Improvement, organism ID 640 (https://www.pulsedb.org/organism/640).
