# Supplementary material for: Genome-wide association mapping of resistant starch for improved nutritional traits in lentils
Source: Front Plant Sci. 2026 Oct 30;17:1937124. doi: 10.3389/fpls.2026.1937124 (PMC13759784; doi:10.3389/fpls.2026.1937124)
Supplement: Supplementary file 1 [file DataSheet1.pdf]

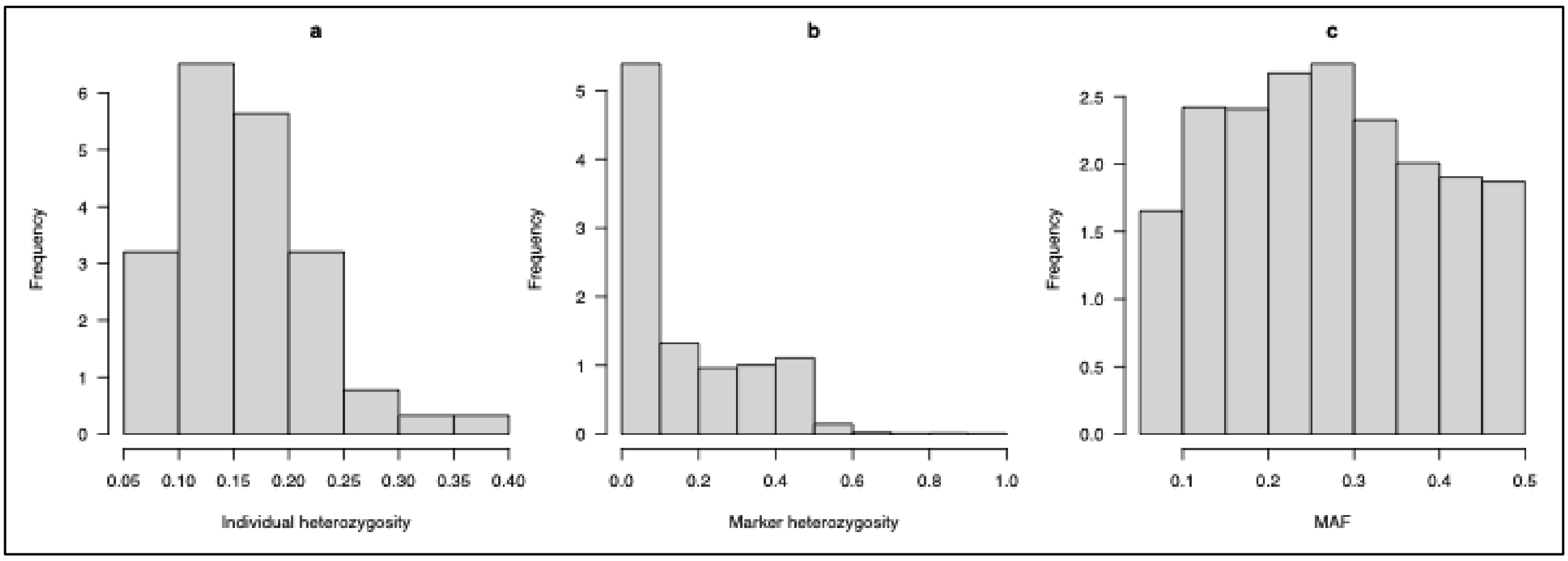

Supplemental Figure 1. GAPIT genotype frequency (a) individual heterozygosity, (b) marker heterozygosity, (c) minor allele frequency (MAF).
