# Supplementary material for: Genome-wide association mapping of resistant starch for improved nutritional traits in lentils
Source: Front Plant Sci. 2026 Oct 30;17:1937124. doi: 10.3389/fpls.2026.1937124 (PMC13759784; doi:10.3389/fpls.2026.1937124)
Supplement: Supplementary file 2 [file Table3.docx]

**Supplementary** **Table 3.** Significance of fixed and random effects from the mixed-model analysis of resistant starch (RS), total starch (TS), 100-seed weight (100SW), and plot seed weight (PlotSW) across three environments. Environment was treated as a fixed effect and tested using an F test. Block, genotype, and genotype × environment interaction were treated as random effects and evaluated using likelihood-ratio tests (LRT). Cullis H²: Generalized broad-sense heritability based on genotype BLUPs.

| Sources | RS | TS | 100SW | PlotSW |
| --- | --- | --- | --- | --- |
| Environment (E) | <0.0001*** | <0.0001*** | <0.0001*** | <0.0001*** |
| Block within environment (B) | <0.0001*** | <0.0001*** | 0.0007** | <0.0001*** |
| Genotype (G) | <0.0001*** | 0.0612 ns | <0.0001*** | <0.0001*** |
| G x E | 0.0660 ns | 0.1376 ns | <0.0001*** | <0.0001*** |
| Cullis H^2^ | 0.63 | 0.18 | 0.98 | 0.44 |

*p<0.01; **p<0.001; ***p<0.0001
